# The influence of peer’s social networks on adolescent’s cannabis use: a systematic review of longitudinal studies

**DOI:** 10.3389/fpsyt.2023.1306439

**Published:** 2023-12-21

**Authors:** María-Carmen Torrejón-Guirado, Miguel Ángel Baena-Jiménez, Marta Lima-Serrano, Hein de Vries, Liesbeth Mercken

**Affiliations:** ^1^Department of Health Promotion, Care and Public Health Research Institute CAPHRI, Maastricht University, Maastricht, Netherlands; ^2^Department of Nursing, Faculty of Nursing, Physiotherapy, and Podiatry, University of Seville, Seville, Spain; ^3^Department of General and Specialist Surgery, Hospital Universitario Virgen Macarena, Seville, Spain; ^4^Department of Health Psychology, Open University of the Netherlands, Heerlen, Netherlands

**Keywords:** peer influence, adolescents, cannabis, social network analysis, friendship

## Abstract

**Aim:**

A systematic review was performed to summarize the key findings of the peer influence on cannabis use through Social Network Analysis (SNA) studies and identify limitations and gaps with the purpose of informing future research and practice. Longitudinal studies were included since they provide robust information about social relationships change over time.

**Background:**

Adolescents’ cannabis use is a global problem, which has awakened an interest in its determinants such as social influences. Research has shown the importance of these influences on cannabis uptake and use. SNA is an useful relational approach to examine socialization mechanisms related to the onset of cannabis use in adolescents.

**Method:**

A search was conducted in PyscINFO, PubMed, Scopus and Web of Science for longitudinal articles published until February 2023, to examine cannabis use and peer’s social networks. We focus on peers’ influence of peers on cannabis use. Additionally, information about effect of cannabis use for peer selection was collected.

**Results:**

The results of the included studies (*n* = 8) showed that friends’ cannabis use was most often/strongly associated with cannabis use. There was also an increase of cannabis use when the adolescent did not feel close to the school’s peers, had a higher proportion of friendships relative to the total number of ties in the neighborhood, had a central position, did not belong to any group but had ties to members of two or more groups, had cannabis user friends (especially in early ages), and lived in a neighborhood where cannabis was used.

**Conclusion:**

Cannabis use is mainly related to friends’ use. Yet, future studies are warranted to control for relevant selection effects to further knowledge on network effects on cannabis use, improving the design, and improving the modeling of the network. This systematic review may inform about the critical aspects of preventing cannabis use among adolescents, taking into consideration their complex social environment.

## 1 Introduction

Cannabis is the third most consumed drug among adolescents (1, 2). According to the World Drug Report issued in 2021 (2), 13.8 million of adolescents aged between 15 and 16 years old used cannabis worldwide. This figure is equivalent to 5.6% of the global population and exceeds the prevalence rate among the general population (3). Furthermore, the European report on drugs (2021) declared cannabis to be the most established drug used in Europe, finding that 15.4% of young people had consumed cannabis in the previous year (1). It is also important to note that cannabis has become a legal drug in many high-income countries in recent decades (4). This legalization of cannabis has led to a 3.8% increase in recreational use in those states (5).

The onset age of cannabis use is around 15 years old (1, 2). Adolescents are particularly vulnerable to cannabis use because their brain is still developing (6). Some consequences of cannabis use are short-term, such as cognition and coordination problems, toxicity or traffic injuries, but also long-term, such as mental disorders, addiction, suicide risk, and cardiovascular or pulmonary diseases (6–8). These consequences, furthermore, can affect the social and family sphere, leading to, for instance, school dropout and family/friend’s problems (e.g., breach of family rules, physical and psychological violence, deterioration, or loss of relationships) (7–9). Moreover, adolescents are sensitive to joining and remaining in peer groups. Peers are individuals who share similar ages and interests, and tend to belong to a similar social group. Adolescents are highly influenced by peers since they try to resolve disagreements by adopting peers’ behaviors (10).

The importance of peer influence on adolescent’s recreational cannabis use has been demonstrated in a substantial body of research (11–13). For instance, peers close to adolescents exert a stronger influence on adolescent cannabis use than peers less close to adolescents (12). Additionally, well-stablished socio-cognitive theories discuss the importance of peer influences on risk behaviors (11–13). A first type of peer influence concerns social modelling. This factor, proposed by Bandura in his Social Cognitive theory (14), implies that behaviors of others such as cannabis use can be adopted by merely observing their behavior (15, 16). When such a behavior is reinforced, this behavior is more likely to become adopted (14). This process may occur unconsciously. Furthermore, occupied social position within a peer group can also play a significant role in how adolescents are influenced toward cannabis use. Adolescents may see high-status peers or more popular peers (usually cannabis users) as role models, in order to improve their own social standing into the group (13). Another type of social influence concerns social norms, a construct originally proposed by Fishbein and Ajzen in the theory of Reasoned Action (17). Norms of other people have shown to influence other persons behavior, including cannabis use such as previous studies have shown regarding adolescent peer’s social norms favoring cannabis use (10, 11). Furthermore, during adolescence, it is expected that youth may not reject the cannabis use offers because they want to fit in with their peers (11). An even more explicit type of social influence concerns direct peer pressure from others (18, 19). These three types of social influence can operate and can have unique contributions (20–23).

The traditional theoretical concepts about social influences did not look to the complex constellation of peer’s influence processes that can modulate the social modelling, norms or pressure (e.g., the influence of social structure of the friendships or the interactions between the adolescents within a social network). Taking such a social network approach implies the use of Social Network Analysis (SNA). SNA is focusing on examining the social structure and interactions among social actors within a social network (24). A network is comprised of nodes (i.e., individuals/actors) and the ties/relationships between those nodes. SNA assumes that social actors and the network they are embedded in are interdependent (25). SNA also makes it possible to control correctly for possible selection and confounding effects while examining peer influence processes, for instance, regarding the cannabis use. Besides, adolescent’s cannabis use can also become similar to their peers because they select each other based on similar cannabis use behavior or due to peers jointly being influenced by an external source (i.e., both watching a movie in which actors use cannabis) (26, 27). This also adds value in regard to previous approaches in cannabis prevention field. Several studies have now applied SNA to examine peer network influences in adolescent cannabis use (28, 29). So it is now timely and relevant to review and summarize their findings.

Longitudinal studies may provide important insights into dynamic social relationships, since they address temporality (30). They consider the dynamics of social phenomena as being the result of a time-process, where observations are made at different time points. Reviewing longitudinal studies is needed in order to understand dynamic relationships between individuals and peer groups patterns regarding cannabis use, and broader patterns of social change over time (31). Thus, this review included only longitudinal studies in order to tracking the influence of peers on cannabis use over time (causal relationship), reducing the bias inherent in cross-sectional studies, and facilitating future comprehensive experimental research.

The goal of this study is to systematically synthesize the scientific literature on longitudinal applications of social network approach to study peer influences on adolescent recreational cannabis use. The research question was: do peer networks characteristics influence in recreational cannabis use in adolescents between 12 and 21 years? This review will summarize the key characteristics and findings of peer influences on recreational cannabis use and identify limitations and gaps in the literature, with the purpose of informing future research and practice. Additionally, measures for selection effects on cannabis use will be also included.

## 2 Methods

### 2.1 Search strategy and retrieval system

For conducting and communicating this systematic review, the recommendations of PRISMA 2020 statement were followed (32). The protocol was pre-registered in PROSPERO (33). Eligible studies were identified in October 2021 and the search was updated in February 2023 by conducting an in-depth literature search on electronic databases used (peer-reviewed): PsycINFO, Scopus, PubMed and Web of Science. The search strategy used a combination of Boolean connections and search terms relevant to key concepts used in this review: social network, cannabis use and adolescent. As example, the specific search strategy in the PubMed database was:

((Network*[tiab]AND friend*[tiab]) OR relation*[tiab] OR peer*[tiab] OR social*[tiab] OR media*[tiab] OR acquaintance*[tiab] OR team*[tiab] OR mate*[tiab] OR partner*[tiab] OR leisure[tiab] OR hobby[tiab] OR school[tiab] OR highschool[tiab] OR university[tiab] OR junior high* OR senior high*[tiab] OR colleague*[tiab]) AND (“Adolescent”[Mesh] OR “Young Adult”[Mesh] OR adolescen*[tiab] OR teen*[tiab] OR young*[tiab] OR youth*[tiab] OR puber*[tiab] OR minor*[tiab] OR juvenil*[tiab] OR student*[tiab] OR pupil*[tiab]) AND (“Marijuana Use”[Mesh] OR “Marijuana Smoking”[Mesh] OR “Cannabis”[Mesh] OR cannabi*[tiab] OR blunt*[tiab] OR marijuana*[tiab] OR marihuana*[tiab])

Additionally, we searched reference lists from each study (backward) and articles that cite back to a specific article (forward), for additional articles. No restrictions were placed on the language and the time window of search.

### 2.2 Study selection criteria

#### 2.2.1 Inclusion criteria

- Study design: longitudinal empirical studies.
- Study subject, target population: adolescents (between 12 and 21 years old). Although organizations as the World Health Organization or American Academy of Pediatric usually define the adolescence as the period of 10–21 years, adolescents from 12 years were chosen because cannabis is a substance which use starts late (34, 35). In case there were studies where they include our age’s rank but also older ages, we will only take the data from our target population, if it is possible.
- Statistical method: Social Network Analysis (SNA) descriptive analysis and/or statistical models for social networks.

#### 2.2.2 Exclusion criteria

- Medicinal cannabis use and patients.
- Intervention studies.
- No collection of complete social network data (i.e., no information on social actors and the ties among them, collection of individual data or only dyad and triad level data).
- Simulation studies that focused on varying intervention parameters.
- Exclusive use of conventional statistical method (e.g., regression) rather than SNA in data analysis.

### 2.3 Data extraction and preparation

The process was developed in the following phases: firstly, after deleting the duplicates, a screening of articles based on title and summary following the inclusion / exclusion criteria was done. The second screening was through the reading of full texts, to which methodological evaluation was also done. This process was done independently by two authors (MCTG and MABJ), who also independently checked results to evaluate inclusion and exclusion criteria. Discrepancies between these two authors were discussed and a final determination decided by a third author (LM). Data were extracted by a structured form. The following variables were considered: instrument, article ID, publication year, country, design, sample, type of network, social network measures, SNA performed, type of software used to conduct the SNA, and main findings.

### 2.4 Data synthesis

The studies were heterogeneous in the types of measures used, and it was therefore more appropriate to follow a narrative data synthesis strategy. The review was focused on variables related to peer social network influences and not on other types of network ties (such as family, social kin or broader social contacts).

All included *peer network influence effects on cannabis use* were extracted from each included manuscript. They were classified in three groups: (1) *Endogenous network influence effects on cannabis use* (the influence of the network structure and interactions itself without considering other variables such as the network’ behavior or personal characteristics), (2) *Cannabis-related network influence effects on cannabis use* (the influence of the network considering the cannabis use of actors in the network) and, (3) *Other risk behavior related network effects on cannabis use* (e.g., alcohol drinking, or personal characteristics). Interaction effects between peer network influence effects were also extracted (e.g., popularity x density).

A few examples of *endogenous network influence effects on cannabis use* are the effect of number of nominated friends (Outdegree); the effect of number of incoming friendship ties/relationships (Indegree/popularity); the effect of being in a central position within the network (centrality).

Examples of *cannabis-related network influence effects on cannabis use* are the effect of cannabis use of someone’s friends (friends’ cannabis use); the effect of number of steps it takes for someone to reach the nearest cannabis user in the network considered friends and friend’s friends (distance to nearest cannabis users considered friends and friend’s friends).

Examples of *other risk behavior related network effects on cannabis use* are the effect of smoking behavior of someone’s friends (friends’ smoking behavior); the effect of the age of someone’s friends (friends’ age).

Additionally, for those studies that controlled for selection effects, *cannabis use related selection effects* were extracted (being the outcome the selection of peers instead of the cannabis use). Examples of cannabis use related selection effects are the effect of own cannabis use on selecting an actor as a friend (cannabis use Ego); the effect of cannabis use of another actor on selecting that actor as a friend (cannabis use alter); the effect of having similar cannabis use to another actor on selecting that actor as a friend (cannabis use similarity).

### 2.5 Study quality assessment

Two reviewers (MCTG and MABJ) independently assessed risk of bias for all studies that met eligibility criteria, using the National Institutes of Health’s Quality Assessment Tool for Observational Cohort, Case–Control Studies, Systematic reviews and Meta-Analyses (36). The assessment tool rates each study based on 14 criteria. For each criterion, a score of one was assigned if ‘yes’ was the response, whereas a score of zero was assigned otherwise (i.e., an answer of ‘no,’ ‘not applicable,’ ‘not reported’ or ‘cannot determine’). The study-specific global score could range from 0 to 14. Discrepancies between MCTG and MABJ were discussed, and a final determination was decided by a third author (LM).

## 3 Results

### 3.1 Study selection

**Figure 1:**
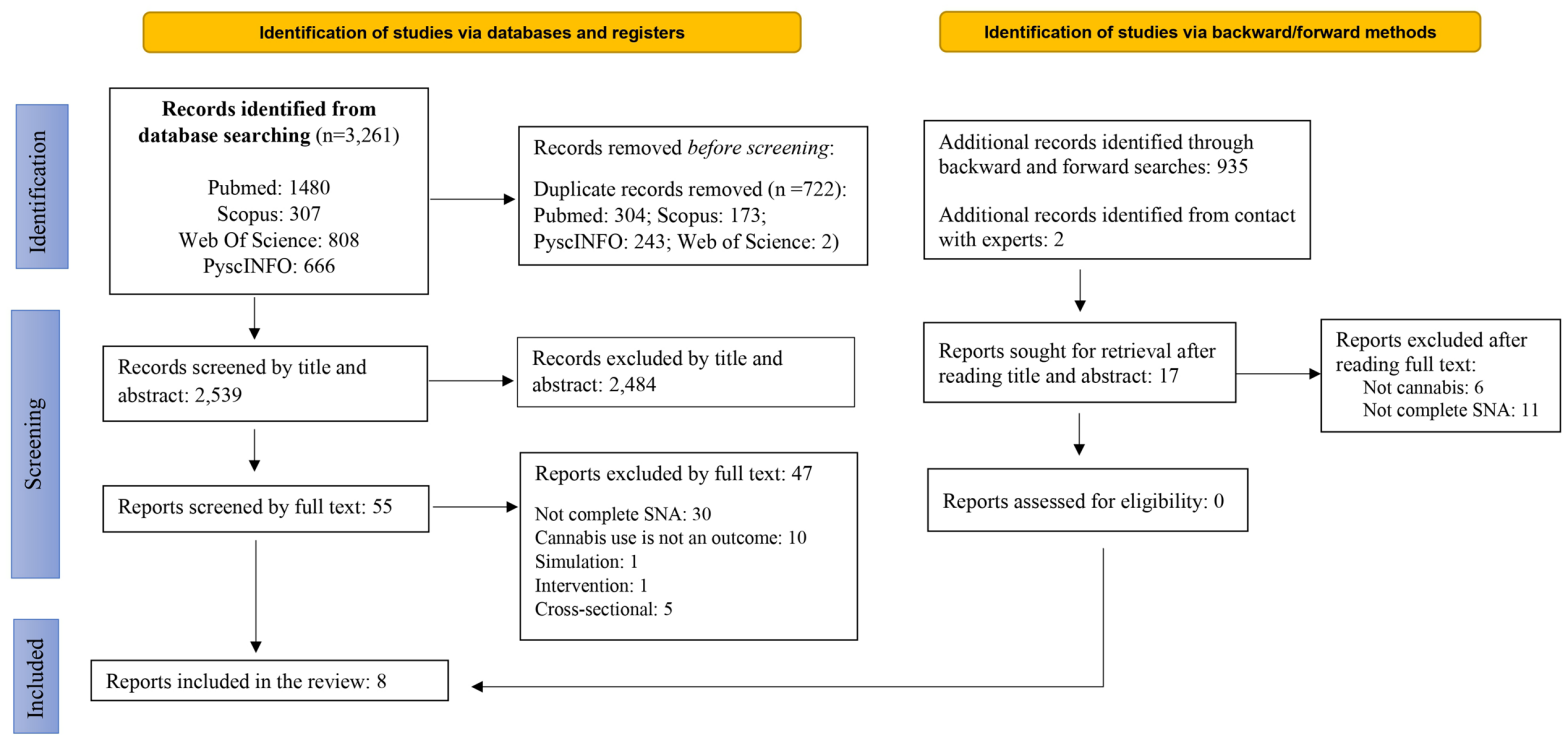
PRISMA 2020 flow diagram for new systematic reviews of reviewed studies.

As depicted in the flow diagram (Figure 1), the initial search yielded 3,261 scientific articles. Adding the articles identified through backward and forward searching, a total of 4,196 articles were found.

During the review process, many studies that had used the traditional regression-based approach to study the influence of peers on cannabis use were found, that is that they estimated peer effect without developing a complete network.

The main reasons for exclusion were that articles focused on medicinal cannabis use, patients, did not use statistical models for social networks such as SNA, did not include a whole or complete social network, were not longitudinal, or did not meet the age criterion (12–21 years). Finally, 8 articles were included for review (37–44).

### 3.2 Study quality assessment

Table 1 reports the specific scoring rubric used for assessing each study’s methodological quality. All included studies had a high-quality level according to the used tool. Each study clearly defined their research question, their target population, their design, and their analytic methods. However, there were three studies that did not control for the most important confounder in peer influence research, namely selection effects, implying that the estimations of these effects may be bias (40, 43, 44). Thus, their results were treated with caution.

**Table 1:** Summary of the criteria used for assessing each study’s methodological quality^36^.

	Wang et al. (2018) (37)	Schaefer (2018) (38)	De la Haye et al. (2015) (39)	Vogel et al. (2015) (40)	Tucker et al. (2014) (41)	De la Haye et al. (2013) (42)	Osgood et al (2014) (43)	Ennett et al. (2006) (44)
1.Was the research question or objective in this paper clearly stated?	Yes	Yes	Yes	Yes	Yes	Yes	Yes	Yes
2.Was the study population clearly specified and defined?	Yes	Yes	Yes	Yes	Yes	Yes	Yes	Yes
3.Was the participation rate of eligible persons at least 50%?	Yes	Yes	Yes	Yes	Yes	Yes	Yes	Yes
4.Were all the subjects selected or recruited from the same or similar populations (including the same time period)? Were inclusion and exclusion criteria for being in the study pre-specified and applied uniformly to all participants?	Not reported	Yes	Yes	Yes	Yes	Yes	Yes	Yes
5.Was a sample size justification, power description or variance and effect estimates provided?	Yes	Yes	Yes	Yes	Yes	Yes	Yes	Yes
6.For the analyses in this paper, were the exposure(s) of interest measured prior to the outcome(s) being measured?	Yes	Yes	Yes	Yes	Yes	Yes	Yes	Yes
7.Was the timeframe sufficient so that one could reasonably expect to see an association between exposure and outcome if it existed?	Yes	Yes	Yes	Yes	Yes	Yes	Yes	Yes
8.For exposures that can vary in amount or level, did the study examine different levels of the exposure as related to the outcome?	Yes	Yes	Yes	Yes	Yes	Yes	Yes	Yes
9.Were the exposure measures (independent variables) clearly defined, valid, reliable, and implemented consistently across all study participants?	Yes	Yes	Yes	Yes	Yes	Yes	Yes	Yes
10.Was the exposure(s) assessed more than once over time?	Yes	Yes	Yes	Yes	Yes	Yes	Yes	Yes
11.Were the outcome measures (dependent variables) clearly defined, valid, reliable, and implemented consistently across all study participants?	Yes	Yes	Yes	Yes	Yes	Yes	Yes	Yes
12.Were the outcome assessors blinded to the exposure status of participants?	Yes	Yes	Yes	Yes	Yes	Yes	Yes	Yes
13.Was loss to follow-up after baseline 20% or less?	Yes	Yes	Yes	Yes	Yes	Yes	Yes	Yes
14.Were key potential confounding variables measured and adjusted statistically for their impact on the relationship between exposure(s) and outcome(s)?	Yes	Yes	Yes	No*	Yes	Yes	No*	No*
Sum Score	13/14	14/14	14/14	13/14	14/14	14/14	13/14	13/14

*No control for selection effects.

### 3.3 Basic characteristics of the included studies

Table 2 summarizes the characteristics of the eight longitudinal studies that met the inclusion criteria. The reviewed studies had been published in six journals that mainly emphasized adolescence. Three of the eight studies had common authors (39, 42, 43). Every study had been conducted in the USA and used at least three data measurement points. The included studies were published between 2006 and 2018. Six of the eight studies used data from the Add Health study (The National Longitudinal Study of Adolescent to Adult Health) (37–42), one study used data from the PROSPER study (Promoting School-Community-University Partnerships to Enhance Resilience) (43), and one had collected its own school network data (44). The sample size ranged from 1,373 to 9,500 participants, and mostly examined adolescents aged 15–16 years old. To focus on friendship ties among students within the same school, each school was defined as a separate social network, and most of the included studies had created a specific model for each school group (37, 39, 41, 42, 44).

**Table 2:** Characteristics of the longitudinal included studies.

Data source (country)	Authors (year)	Study details	Outcome	Type of network(s): (friendship ties, family ties, best friend ties, etc)	Social network measure(s) (Peer influence effects on cannabis use and peer selection effects related to cannabis use)	Social network analysis, software and built models
Add Health (USA)	Wang et al. (2018) (37)	3 waves *n* = 3,128 Aged 12–17 48% female	Cannabis last month into 3 levels: 0 = “never,”1 = “1–10times,”2 = “more than 10 times”	2 school friendship networks Friendship ties: nominations of maximum 5 male best friends and 5 female best friends, within the same school (not necessarily the same grade).	Peer Influence effects on cannabis use: Indegree (own popularity; received friendship nominations) Peer influence cannabis use Number of friends who smoked Number of friends who drank Peer Selection effects on cannabis use: Similarity of cannabis use	SNA (SABM)/R Siena For each school, three-wave SAB model was built. Each model had three behaviour functions, each modelling the dynamic of one behaviour: alcohol use, tobacco use and cannabis use. In this review, we just focused on cannabis. In the behavior equations, peer influence effects were measured as the sum of negative absolute difference between ego’s and alters’ behavior averaged by ego’s out-degree. In the network equation, they included endogenous network effects (e.g., reciprocity) and homophily selection effects for each substance use behavior as well as additional covariates.
	Schaefer (2018) (38)	3 waves *n* = 1,373 Mean age: 15.56 48.87% female	Cannabis last month was recoded into dichotomous measure (1 = yes cannabis use, 0 = no cannabis use)	2 school friendship networks Friendship ties: nominations of maximum 5 male best friends and 5 female best friends, within the same school (not necessarily the same grade).	Peer Influence effects on cannabis use: Friend’s cannabis use (i.e., average similarity of cannabis use, that is, adolescent’s preference to have similar cannabis use than their friends, where the total influence of the friends is the same regardless of the number of friends) Peer Selection effects on cannabis use: Ego’s cannabis use Alter’s cannabis use Similarity cannabis use Ego’s cannabis use x R alter R ego x alter’s cannabis use *R refers to ego’s risk factors	SNA (SAOM)/ R Siena Four R Siena models were built for both schools. The difference between the models is that each model provided an illustration of the SAOM for one risk factor of cannabis use. Risk factors are: M1 = Family Connectedness, M2 = School belonging, M3 = Grade point average (GPA), M4 = Religiosity and M5 = Self-control. Changes in the friendship network were modelled with two functions: a rate function that determines which actor is given the chance to change a tie, and a friend selection function that determines which change a chosen actor makes. Change in a given behavior was modeled with two functions: a rate function to specify how often individuals are given the chance to change their behavior, and a behavior function that includes predictors of behavior change. They used a multigroup method to estimate one pooled model for the two schools.
	De la Haye et al. (2015) (39)	3 waves *n* = 1,612 Mean age: 16.4 48.87% female	A dichotomous measure of lifetime of cannabis use was computed at each wave where 1 = ever used cannabis, with changes from 0 to 1 in history of use between waves capturing cannabis initiation.	16 school friendship networks Friendship tie: nominations of maximum 5 male best friends and 5 female best friends, within the same school grade.	Peer Influence effects on cannabis use: Friends lifetime cannabis use Peer Selection effects on cannabis use: Any history of cannabis use and current cannabis use of ego Any history of cannabis use and current cannabis use of alter Same history or same current cannabis use	SNA (SABM)/R siena 4.0 For each school, various R Siena models were built. Baseline models (M1) included effects of history of cannabis use and covariates, but not risk factors, in predicting the friendship network and history of cannabis use. Phase 2 models (M2) added effects of current (last month) cannabis use on friendship choices. Phase 3 models tested for effects of each of the risk factors on friendship choices. Phase 3 included all parameters from M2 model, + the three new effects (ego, alter, similar) of the risk factor on friendship selection, and the effect of the risk factor on change in history of cannabis use (i.e., cannabis initiation). Final models (M3) included risk factors that were found to significantly predict friendship choices and/or history of marijuana use in phase 3 alongside parameters included in M1 and M2. Therefore, M1 = friendship network and history of cannabis use dynamics; M2 = friendship network, history of cannabis use, and current cannabis use dynamics; M3 = friendship network, cannabis use, and risk factor dynamics. Only significant effects on friendships or cannabis initiation, independent of other risk factors, were retained in M3.
	Vogel et al. (2015) (40)	3 waves *n* = 7,754 Mean age: 15.2 55% female	Cannabis last month was used as dichotomous measure (1 = yes cannabis use, 0 = no cannabis use)	109 school friendship networks Friendship tie: nominations of maximum 5 male best friends and 5 female best friends, within the same school grade.	Peer Influence effects on cannabis use: Own popularity (indegree; number of friendship nominations an individual received on the friendship roster) Peer substance use (alcohol and tobacco) Network centrality (the number of ties the respondent has weighted by the number of ties of those to whom he/she sends and received nominations) Popularity x density; popularity x school connectedness; popularity x school drug use (*drug use is referred to alcohol and tobacco*)	Multilevel logistic regression models on Stata/preconstructed measures from secondary data that can be found on the Add health website. Three hierarchical logistic regression models were performed: model 1 included the main effects of the socio-demographic characteristics, individual risk factors, and peer-network characteristics on self-reported cannabis use. Second model introduced the school level covariates (network density, connectedness, and normative drug culture) and model 3 included three interactions of school context (popularity x density, popularity x connectedness, popularity x school drug use).
	Tucker et al. (2014) (41)	3 waves *n* = 1,612 Mean age: 16.4 47.3% female	Cannabis last month into 4 levels: 0 = none, 1 = 1–3 times, 2 = 4–11 times, 3 = 12–32 times, and 4 = 33 times or more.	2 schools friendship networks Friendship tie: nominations of maximum 5 male best friends and 5 female best friends, within the same school grade.	Peer Influence effects on cannabis use: *These effects were measured through interactions (*e.g.*, friend’s cannabis use x reciprocity).* Friends’ cannabis use Reciprocity (both participants nominated each other as a friend) Friend popularity (friend indegree: the total number of friendship nominations received by a nominated friend) Popularity difference of respondent (the difference in number of friend nominations received, i.e., indegree) Peer Selection effects on cannabis use: Ego’s cannabis use Alter’s cannabis use Squared alter cannabis use Same cannabis use	SNA - SABM/R Siena For each school, 3 R Siena models were build. The only difference between the 3 models is that each one of the models included one specific interaction with peer influence, either friends’ cannabis use (influence) × Friendship reciprocity (model 1-M1), Friends’ cannabis use (influence) × friend popularity (model 2-M2) or Friends’ cannabis use (influence) × popularity difference (model 3-M3).
	De la Haye et al. (2013) (42)	3 waves *n* = 1,612 Mean age: 16.4	Cannabis last month into 4 levels: 0 = none, 1 = 1–3 times, 2 = 4–11 times, 3 = 12–32 times, and 4 = 33 times or more. They also created a dichotomous measure of lifetime use at each wave (where 1 = had ever used cannabis, with changes from 0 to 1 in lifetime use between waves capturing initiation)	2 school friendship networks Friendship tie: nominations of maximum 5 male best friends and 5 female best friends, within the same school grade and out-school friends, but finally they only included friends were also survey respondents	Peer Influence effects on cannabis use: Friends’ cannabis use (last month/lifetime) Peer Selection effects on cannabis use: Ego’s cannabis use Alter’s cannabis use Squared alter cannabis use (on last month) Same cannabis use	SNA, SABM/R Siena Two R Siena models were estimated to examine associations of adolescent friendships with (1) cannabis initiation, and (2) frequency of past month cannabis use. Each model includes effects predicting the evolution of the friendship network (friend selection effects) and effects predicting cannabis use (cannabis effects). For cannabis initiation, friend influence was tested with two effects: the effect of having friends who had ever used cannabis in their lifetime, and the effect of having friends who had used cannabis in the past month.
PROSPER (Promoting School-Community-University Partnerships to Enhance Resilience) (USA)	Osgood et al. (2014) (43)	5 waves *n* = 9,500 Mean age: 6th through 9th grades 51.5% female	Cannabis last month into 5 levels: from 1 = “Not at all” to 5 = “More than once a week.”	27 school friendship networks	Peer Influence Effects: Indegree (own popularity; friendship nominations an individual received, i.e., number of other students who named the respondent)	Multi-level logistic regression model/a routine programmed themselves (SAS)
				Friendship tie: nominations of maximum 2 best friends and 5 additional friends from their current school grade. 368 school grade cohort friendship networks.	“Group members” were distinguished as (1) core member vs. (2) peripheral members of the group (meaning that removing a single friendship link would be sufficient to separate them from the main portion of the group), while “non-group members” are (1) isolates (who did not send or receive friendship nominations to anyone else in the grade network or who shared ties with one person who was disconnected from the rest of the network, forming an isolated dyad), (2) liaison (who had ties to members of two or more groups) and (3) other non-members (students who were not defined as members of a group, a liaison, or an isolate). All participants are core group members except those labelled as peripheral group members, liaison and isolate. Core member was treated as the reference to test the others. Friend’s cannabis use Outdegree (number of friendship nominations an individual made) Reach (reach of an adolescent to others in the networks through pathways of ties)	A three-level logistic regression was performed. Five waves of data (level 1) as nested within individual respondents (level 2-stable individual differences in substance use), who are in nested within the school district cohorts that define the social networks (level 3-unexplained differences among social networks in rates of substance use). For the group detection they used a variant of Moody’s CROWDs routine, which is similar in form to other algorithms designed to search for groups by maximizing modularity scores.
Context of Adolescent Substance Use Study (USA)	Ennett et al. (2006) (44)	5 waves *n* = 5,104 Mean age: sixth (35.9%), seventh (33.1%), and eighth (31.0%) graders 65.5% female	Cannabis last 3 months: from 0 to 10 or more times. *Because responses were skewed toward never and infrequent use, a binary variable was formed for each that contrasted adolescents who reported any days/times of use in the last 3 months with those who reported none.	26 separate networks from 13 schools. Friendship tie: nominations of maximum 5 best friend within the same school grade.	Peer influence effects: Social embeddedness: Reciprocity Neighborhood density (number of friendship ties present among friends/alters divided by the total number of possible ties) Out nominations (out-degree)	Three-level hierarchical generalized linear models/SAS IML was used to calculate all measures except two: betweenness centrality and Bonacich power centrality, which were calculated by UCINET (Version 6).
					Social position: (1) Group member (who shared most of their friendship ties with each other and where the removal of one member of the group would not cause the group to be disconnected, (2) Isolate (one or no friendship ties) and (3) Bridge (those with friendship ties to adolescents who were members of different groups, but who were not themselves members of any group) *The three social positions were measured by two dummy-coded variables with group member as the reference Social status: Normed indegree (own popularity, measured by number of friendship nominations received by ego divided by the number of possible friendship nominations) Reach centrality (incoming ties only) Betweenness centrality (the possibility to the adolescent can control flows of information or norms by serving as a gatekeeper between peers, and can connect peers from different parts of the network who are not directly connected to each other) Bonacich power centrality (centrality of the friends with whom ego is linked) Social proximity to cannabis users: Best friend cannabis user (having a best friend who reported recent use) No. neighborhood cannabis users Distance to cannabis user (low coding for nearest user friends or nearest user friend’s friends)	A three-level hierarchical generalized linear model was performed. The three levels were time nested within adolescents nested within networks. Data were arranged in a cohort sequential design with adolescent age. For each network variable, they presented the exponentiated b coefficient predicting the starting point of cannabis use at the different ages (i.e., the age 11 odds ratio). They included the main effect of the network variable and the interaction between the network variable and age. Moody’s CROWDS algorithm for identifying peer groups was also used to measure adolescents’ group position in the network as a group member, bridge, or isolate.

*Means relevant clarifications.

Table 2 also reports the outcome measures, social network characteristics, and statistical models used. Cannabis use in the last 3 months was the outcome of one study (44), cannabis use in the last month was the outcome of the remaining seven studies (37–43).

In all studies, social network data were constructed based on nominations within school grades, provided by respondents via survey data. The studies differed in the type of nominations and the maximum allowed number of nominations. Regarding the number and the type of allowed nominations, six studies asked respondents to nominate ten of their best friends (five females and five males) (37–42), one study asked to nominate two best friends and five additional friends (42)^,^ and the last one asked to nominate a maximum of five best friends (44). The last studies did not make a distinction by gender. Regarding the social network methods used, five studies applied stochastic actor-based/orientated models (SABM/SAOM) via R Siena (37–39, 41, 42), one of them used network measures that was already provided on Add Health (39), one used their own network analysis routine (43), and the last one calculated network measures in UCINET that were later treated as variables in three-level hierarchical generalized linear models (44).

### 3.4 Key findings regarding peer network influence effects on cannabis use

The main objectives of the study and key findings about peer network influences on adolescent cannabis use are presented in Table 3. Furthermore, the definitions of the included effects (Supplementary Table S1) and a more concise overview of the significance of all included peer influence (Supplementary Table S2) are available in the supplementary materials.

**Table 3:** Aim and major findings related to peer influence and selection effects on adolescent’s cannabis use.

Data Source	Authors (year)	Objective	Synthesis of results: Major findings related to the social network analyses
Add Health	Wang et al. (2018) (37)	To examine the co-evolution of adolescent friendship network ties and whether there was interdependence in usage of cigarettes, alcohol, and cannabis	Peer influence effects on cannabis use: School 1: In-degree (own popularity) (*β* = 0.03, *p* > 0.05) Cannabis use peer influence (***β* = 1.43, *p* < 0.01**) Number of friends who smoked (*β* = 0.02, *p* > 0.05) Number of friends who drank (*β* = −0.04, *p* > 0.05) School 2: In-degree (own popularity) (*β* = 0.02, *p* > 0.05) Cannabis use peer influence (***β* = 1.32, *p* < 0.001**) Number of friends who smoked (*β* = 0.03, *p* > 0.05) Number of friends who drank (*β* = −0.04, *p* > 0.05) Peer selection effects on cannabis use: School 1: Similarity cannabis use:***β* = 0.27, *p* < 0.001**. School 2: Similarity cannabis use on school 2:***β* = 0.22, *p* < 0.01**.
	Schaefer (2018) (38)	The aim was in the systematic network selection processes that lead adolescents into friendships with substance-using peers	Peer influence effects on cannabis use: Friend’s cannabis use (average similarity): M1**(*β* = 2.122, *p* < 0.001);** M2**(*β* = 2.029, *p* < 0.01);** M3**(*β* = 1.887, *p* < 0.05)**; M4**(*β* = 2.338**,***p* < 0.001)**; M5**(*β* = 2.184, *p* < 0.001)** Peer selection effects on cannabis use: Cannabis ego: M1 (*β* = −0.24, *p* > 0.05); M2 (*β* = −0.48, *p* > 0.05); M3 (*β* = −0.048, *p* > 0.05); M4 (*β* = −0.029, *p* > 0.05); M5 (*β* = −0.107, *p* > 0.05). Cannabis alter: M1 (*β* = 0.20, *p* > 0.05); M2 (*β* = 0.163, *p* > 0.05); M3 (*β* = 0.207, *p* > 0.05); M4 (*β* = 0.143, *p* > 0.05); M5 (*β* = 0.12, *p* > 0.05). Cannabis similarity: M1 (***β* = 0.395, *p* < 0.01**); M2 (***β* = 0.268, *p* < 0.05**); M3 (***β* = 0.321, *p* < 0.05**); M4 (***β* = 0.395, *p* < 0.001**); M5 (*β* = 0.267, *p* > 0.05). Ego’s cannabis use x R alter: M1 (*β* = −0.108, *p* > 0.05); M2 (***β* = −0.512, *p* < 0.05)**; M3 (*β* = −0.132, *p* > 0.05); M4 (*β* = −0.337, *p* **<** 0.1); M5 (*β* = −0.291, *p* > 0.05). R ego x alter’s cannabis use: M1 (*β* = 0.199, *p* > 0.05); M2 (*β* = −0.269, *p* > 0.05); M3 (*β* = 0.046, *p* > 0.05); M4 (*β* = 0.384, *p* **<** 0.1); M5 (*β* = −0.567, *p* **<** 0.1).
	De la Haye et al. (2015) (39)	The current study tests whether the observed tendency for adolescents to select friends with similar histories of marijuana use (42) is explained by friends’ selection on other risk factors associated with substance use	Peer influence effects on cannabis use: School 1: Friends’ lifetime cannabis use: M1: (**PE = 0.52, *p* < 0.01**); M2: (**PE = 0.52, *p* < 0.01**); M3: (**PE = 0.50, *p* < 0.01**) School 2: Friends’ lifetime cannabis use: M1: (PE = 0.24, *p* > 0.05); M2: (PE = 0.24, *p* > 0.05); M3: (PE = 0.10, *p* > 0.05) Peer selection effects on cannabis use: School 1: Any history of cannabis use ego: M1 (PE = –0.16, *p* > 0.05); M2 (PE = –0.05, *p* > 0.05); M3 (PE = 0.00, *p* > 0.05) Any history of cannabis use alter: M1 (PE = –0.10, *p* > 0.05); M2 (PE = –0.10, *p* > 0.05); M3 (PE = 0.01, *p* > 0.05) Same history of cannabis use: M1 (**PE = 0.27, *p* < 0.01**); M2**(PE = 0.20, *p* < 0.05**); M3 (**PE = 0.18, *p* < 0.05**) Current cannabis use ego: M2 (PE = –0.12, *p* > 0.05); M3 (PE = –0.08, *p* > 0.05) Current cannabis use alter: M2 (PE = 0.06, *p* > 0.05); M3 (PE = 0.11, *p* > 0.05) Same current cannabis use: M2 (**PE = 0.17, *p* < 0.01**); M3 (**PE = 0.16, *p* < 0.01**) School 2: Any history of cannabis use ego: M1 (**PE = –0.27, *p* < 0.01**); M2 (**PE = –0.23, *p* < 0.05**); M3 (PE = –0.23, *p* > 0.05) Any history of cannabis use alter: M1 (PE = 0.14, *p* > 0.05); M2 (PE = 0.15, *p* > 0.05) M3 (PE = 0.12, *p* > 0.05) Same history of cannabis use: M1 (**PE = 0.32, *p* < 0.01**); M2 (**PE = 0.33, *p* < 0.01**); M3 (**PE = 0.30, *p* < 0.01**) Current cannabis use ego: M2 (PE = –0.06, *p* > 0.05) Current cannabis use alter: M2 (PE = –0.06, *p* > 0.05); M3 (PE = 0.12, *p* > 0.05) Same current cannabis use: M2 (PE = –0.02, *p* > 0.05)
	Vogel et al. (2015) (40)	To examine the moderating influence of school connectedness, school drug culture, and global network density on the association between peer network status and cannabis use.	Peer influence effects on cannabis use: Own popularity: M1 (***β* = 0.04, OR = 1.04, CI95% = 1.02, 1.07, *p* < 0.05**), M2 (***β* = 0.04, OR = 1.04, CI95% = 1.01, 1.07, *p* < 0.05**); M3 (***β* = 0.04, OR = 1.04, CI95% = 1.02, 1.07, *p* < 0.05**) Peer substance use (alcohol and tobacco): M1 (***β* = 0.64, OR = 1.90, CI95% = 1.70, 2.13, *p* < 0.001**), M2 (***β* = 0.64, OR = 1.90, CI95% = 1.70, 2.12, *p* < 0.001**); M3 (***β* = 0.64, OR = 1.90, CI95% = 1.70, 2.12, *p* < 0.001**) Network centrality: M1 (*β* = −0.10, OR = 0.90, CI95% = 0.78, 1.04, *p* > 0.05), M2 (*β* = −0.09, OR = 0.91, CI95% = 0.79, 1.05, *p* > 0.05); M3 (*β* = −0.10, OR = 0.91, CI95% = 0.79, 1.04, *p* > 0.05) Own popularity x density: M3 (*β* = <0.00, OR = 0.99, CI95% = 0.97, 1.02, *p* > 0.05) Own popularity x school connectedness: M3 (***β* = −0.05, OR = 0.95, CI95% = 0.91, 0.98, *p* < 0.05**) **·** Own popularity x school drug use (alcohol & tobacco): M3 (*β* = <0.00, OR = 0.99, CI95% = 0.99, 1.01, *p* > 0.05)
	Tucker et al. (2014) (41)	To examine whether structural features of friendships moderate friends’ influence on adolescent cannabis use over time.	Peer Influence effects on cannabis use: School 1: Friends’ cannabis use: M1: not significant*; M2: not significant*; M3: PE = 0.85, *p* = 0.069. Friends’ cannabis use (influence) × friendship reciprocity M1:**PE = 1.14, *p* = 0.028** Friends’ cannabis use (influence) × friend popularity M2: PE = 0.12, *p* = 0.189 Friends’ cannabis use (influence) × popularity difference M3: PE = −0.02, *p* = 0.500 School 2: Friends’ cannabis use: M1: not significant*; M2: not significant*; M3: PE = 0.53, *p* = 0.109 Friends’ cannabis use (influence) × friendship reciprocity M1: PE = 0.51, *p* = 0.254 Friends’ cannabis use (influence) × friend popularity M2:**PE = 0.15, *p* = 0.041** Friends’ cannabis use (influence) × popularity difference M3: PE = 0.01, *p* = 0.709 Note: reciprocity, friend popularity and popularity difference were not measured individually: they were measured in interactions. Peer Selection effects on cannabis use: School 1 Ego’s cannabis use M1: PE = −0.01, *p* = 0.860; M2: PE = −0.01, *p* = 0.905; M3: PE = −0.02, *p* = 0.909 Alter’s cannabis use M1: PE = −0.19, *p* = 0.652; M2: PE = −0.27, *p* = 0.684; M3: PE = −0.26, *p* = 0.873 Squared alter cannabis use M1: PE = 0.14, *p* = 0.314; M2: PE = 0.16, *p* = 0.444; M3: PE = 0.16, *p* = 0.764 Similar/same cannabis use M1:**PE = 1.53, *p* = 0.003;** M2:**PE = 1.49**,***p* = 0.000**; M3:**PE = 1.49**,***p* = 0.004** School 2 Ego’s cannabis use PE = 0.10, *p* = 0.102; M2: PE = 0.09, *p* = 0.103; M3: PE = 0.09, *p* = 0.049 Alter’s cannabis use**PE = 0.49**,***p* = 0.040**; M2: PE = 0.37, *p* = 0.064; M3: PE = 0.39, *p* = 0.071 Squared alter cannabis use PE = 0.41, *p* = 0.195; M2: PE = −0.09, *p* = 0.267; M3: PE = −0.10, *p* = 0.282 Similar/same cannabis use**PE = 1.03**,***p* = 0.000**; M2:**PE = 1.04**,***p* = 0.000**; M3:**PE = 1.02**,***p* = 0.000**
	De la Haye et al. (2013) (42)	(A) To determine the extent to which friendship networks influence cannabis use (influence effects) and cannabis use influences friendship selection (selection effects). (B) to assess if a multiplicative model of risk explains differences in cannabis-based selection and influence.	Peer Influence effects on cannabis use: On lifetime (M1) School 1: Friends’ cannabis use lifetime: not significant* Friends’ cannabis use last month:**PE = 1.31**,***p* = 0.001** School 2 Friends’ cannabis use lifetime: not significant* Friends’ cannabis use last month: PE = 0.61, *p* = 0.116 On last month (M2) School 1 Friend’s cannabis use: PE = 0.63, *p* = 0.126 School 2 Friend’s cannabis use: PE = 0.51, *p* = 0.125 Peer Selection effects on cannabis use: On lifetime (M1)
			School 1 Ego’s cannabis use:**PE = −0.15**,***p* = 0.049** Alter’s cannabis use: PE = −0.10, *p* = 0.099 Same cannabis use: **PE = 0.27**,***p* = 000** School 2 Ego’s cannabis use (lifetime):**PE = −0.14**,***p* = 0.032** Alter’s cannabis use (lifetime): PE = 0.11, *p* = 0.104 Same cannabis use (lifetime):**PE = 0.43**,***p* = 0.000** On last month (M2) School 1 Ego’s cannabis use: PE = −0.02, *p* = 0.822 Alter’s cannabis use: PE = −0.25, *p* = 0.558 Squared alter cannabis use: PE = 0.16, *p* = 0.268 Same cannabis use:**PE = 1.49**,***p* = 0.000** School 2 Ego’s cannabis use: PE = 0.10, *p* = 0.069 Alter’s cannabis use: PE = 0.39, *p* = 0.238 Squared alter cannabis use (past month): PE = −0.09, *p* = 0.459 Same cannabis use:**PE = 1.02**,***p* = 000**
	Osgood et al. (2014) (43)	To examine the association of substance use with the types of positions adolescents hold in cohesive peer groups within the friendship networks of their schools’ grade-cohort.	Peer Influence effects on cannabis use: Indegree (own popularity): (Coef = 0.013, *p* > 0.05). Friendship group position (core member as the reference group): Peripheral member (Coef = 0.112, *p* > 0.05). Isolate (Coef = 0.142, *p* > 0.05). Liaison (**Coef = 0.312, *p* < 0.01**). Non-member (Coef = 0.155, *p* > 0.05). Friend’s cannabis use: Friends’ mean use (**Coef = 3.763, *p* < 0.01**). Friends’ mean use × total simple number of friends (**Coef = 2.408, *p* < 0.01**). Out-degree: (**Coef = −0.125, *p* < 0.01**). Reach: (**Coef = 0.008, *p* < 0.01**).
Context of Adolescent Substance Use Study	Ennett et al. (2006) (44)	To examine the peer context of adolescent substance use, social network analysis was used to measure three domains of attributes of peer networks: social embeddedness, social status, and social proximity to substance users.	Peer influence effects on cannabis use: Social embeddedness: Reciprocity: ages 11 (OR = 0.55, *p* > 0.05); ages 13: (OR = 0.75, *p* > 0.05); ages 15: (OR = 1.01, *p* > 0.05). Neighbourhood density: ages 11 (OR = 0.49, *p* > 0.05); ages 13: (OR = 0.24, *p* > 0.05); ages 15: (**OR = 0.12, *p* < 0.001**). Out nominations (out-degree): ages 11 (OR = 1.28, *p* > 0.05); ages 13: (**OR = 1.26, *p* < 0.05**); ages 15: (**OR = 1.25, *p* < 0.01**). Social position (group member is the reference group): Isolate: ages 11 (OR = 1.11, *p* > 0.05); ages 13: (OR = 0.96, *p* > 0.05); ages 15: (OR = 0.83, *p* > 0.05). Bridge: ages 11 (OR = 1.00, *p* > 0.05); ages 13: (OR = 0.98, *p* > 0.05); ages 15: (OR = 0.96, *p* > 0.05). Social Status: Normed indegree: ages 11 (**OR = 1.66, *p* < 0.05**); ages 13: (**OR = 1.35, *p* < 0.001)**; ages 15: (OR = 1.09, *p* > 0.05) Reach centrality: ages 11 (**OR = 1.05, *p* < 0.05**); ages 13: (**OR = 1.04**,***p* < 0.001**); ages 15: (**OR = 1.02, *p* < 0.05**) Betweenness centrality: ages 11 (OR = 1.07, *p* > 0.05); ages 13: (OR = 1.06, *p* > 0.05); ages 15: (OR = 1.06, *p* > 0.05) Bonacich power centrality: ages 11 (OR = 1.00, *p* > 0.05); ages 13: (OR = 1.01, *p* > 0.05); ages 15: (**OR = 1.03**,***p* < 0.01**). Social proximity to cannabis users: Best friend cannabis user: ages 11 (**OR = 23.34, *p* < 0.001**); ages 13: (**OR = 7.82, *p* < 0.001**); ages 15: (**OR = 2.62, *p* < 0.01**). No. neighbourhood cannabis users: ages 11 (**OR = 7.39, *p* < 0.001**); ages 13: (**OR = 3.77, *p* < 0.001**); ages 15: (**OR = 1.93, *p* < 0.001**) Distance to cannabis user: ages 11 (**OR = 0.25, *p* < 0.001**); ages 13: (**OR = 0.41**,***p* < 0.001**); ages 15: (**OR = 0.66, *p* < 0.001**).

* Authors did not provide the exact value. R, risk factors of 4 attributes: family connectedness, school belonging, grade point average (GPA), religiosity and self-control. PE, parameter estimate; OR, odds ratio. See last column from Table 2 for understanding which models are M1, M2, M3 in each article. Significant of bold values.

#### 3.4.1 Endogenous network influence effects on cannabis use

The most examined effect was own popularity (37, 40, 43, 44). Two of the studies did not find a significant effect (37, 43), while two others found a significant positive effect indicating that the more incoming ties one had, the more cannabis they used (40, 44). One study examined the effect of their own popularity on cannabis use in three different age groups (44), and found that when age increase, the effect of their own popularity decreases. Finally, one study examined whether the effect of own popularity on cannabis use could be moderated by density, school level’s alcohol and tobacco use, and school connectedness (40). Only the interaction with school connectedness was statistically significant, indicating that the effect of own popularity on cannabis use was less strong for schools where students felt happy and close to the people at school (40).

Outdegree, neighborhood density, and reciprocity were jointly examined in one study (44). Although adolescents nominating a higher number of friends (high outdegree) had a significantly higher risk of cannabis use, being nested in denser network neighborhoods protected against cannabis use. Reciprocity did not appear to significantly influence cannabis use (44). However, in this study, possible selection effects were not modelled.

Three studies examined the effect of being in a central position within the network on cannabis use (40, 43, 44). Although different centrality measures were modelled, most centrality effects were not significant (40, 44). Only reach centrality significantly influenced cannabis use in two studies (43, 44). This indicates that adolescents that are closer to all other actors in the network, have a higher tendency to use cannabis in comparison with those who are less close to others. Unfortunately, none of the studies controlling for selection included centrality effects in their models. The same applies for group position effects that were also only examined in two studies not controlling for selection effects (43, 44). Only being a liaison compared to being a core member significantly increased cannabis use (42). These two studies which included influence effects related to the adolescent’s position in the network created different dummy variables to make the comparison (43, 44). The first study distinguished between one cohesive group (core and peripheral members), multiple groups (liaisons), or no group (isolates and other non-members). They were represented through a set of dummy variables that contrast all other positions to core members (43). The second study measured adolescents’ position in the network as a group member, bridge, or isolate. They were measured by two dummy-coded variables with group member as the reference (44). See Supplementary material for more information.

#### 3.4.2 Cannabis-related network influence effects on cannabis use

The cannabis use of friends was included in six studies (37–39, 41–43). Three of them showed a positive significant influence of friends’ cannabis use on adolescent cannabis use (37, 38, 43), while one study showed no significant results (41). The two remaining studies (39, 42) showed mixed results, and one of them had two different outcomes that gave different results (42).

In the first study, adolescent’ lifetime cannabis use was significant influenced by friend’s lifetime cannabis use only at one school (39). In the second study, adolescent’s lifetime cannabis use was not statistically significantly influenced by friend’s lifetime cannabis use. Yet, it was significantly influenced by friend’s last month cannabis use at one school but not the other (42). Additionally, no significant results of friend’s cannabis use were found for adolescent’s last month cannabis use (42).

Only one study modelled the specific effect of social proximity with a cannabis user, where not having a best friend cannabis user, having fewer neighborhood cannabis users, and being at a greater distance to the nearest user among friends and friends’ friends, led to less adolescent cannabis use (44). The influence of best friend’s use was greatest at early adolescence (44).

One study found that the higher mean of friends’ cannabis users, the more adolescent’s cannabis use (43). This study also examined whether the effect of friends’ cannabis use could be moderated by the number of friends. The effect of the average of friends’ users is stronger on own cannabis use in case of lower number of friends, possibly due to the fact that adding a single friend is more consequential for adolescents with fewer friends than for those with many friends (43). Another study examined whether friends’ cannabis use could be moderated by reciprocity, friends’ popularity and popularity difference in two schools (41). The interaction with reciprocity was significant only in one school. In this, the effect of friend’s cannabis use on adolescent’ cannabis use was stronger for adolescents who tended to adopt the cannabis use behaviors of their mutual friends. The interaction with friend popularity was significant also in one of both schools, where adolescents were likely to adopt the cannabis use behaviors of their more popular friends (41).

#### 3.4.3 Other effects of network-related risk behavior related network effects On cannabis use

One study found that adolescents were more likely to use cannabis if their peers used alcohol or tobacco (40). Yet, another study found that the number of friends who smoke and drink at school was not statistically significant with cannabis use (37).

### 3.5 Findings regarding cannabis use related selection effects

There were five SIENA studies that incorporated a co-evolution approach, in which peer influence was controlled for selection effects. Furthermore, they incorporated alternative network and behavior change mechanisms to avoid misdiagnosis of selection or influence effects when another social process is operating. In all five studies, adolescents had the significant tendency to select friends based on similarities in cannabis use behavior, regardless of the outcome (lifetime cannabis use or last month use) and regardless of which operationalization was used for the selection effects in the estimation procedure (37–39, 41, 42). Additionally, cannabis users tended to make fewer friends (39, 42).

## 4 Discussion

This review is the first to longitudinally examine the influence of peer social networks on cannabis use, taking a social network approach. During the review process, the methodological quality of the included studies was critically evaluated, considering the high quality of those studies that controlled for selection effects (37–39, 41, 42). This review found multiple outcomes that sometimes result in different results, such as, for instance, some studies controlling for selection effects and others not.

The most often found effect was the positive influence of friend cannabis use on adolescent cannabis use, meaning that adolescents are more likely to use cannabis if their close friends use cannabis. Yet, different operationalizations were used for assessing the influence of friends’ cannabis use. Other studies not included in this review, have also shown that the influence of peers is an important factor in cannabis use (11, 21, 22, 45). Although these studies did not take a network approach, they showed that adolescents are influenced by the perceived cannabis use of friends (modeling) (21, 22, 39), norms regarding cannabis use among Friends (11, 21, 22), and that they may experience pressure to conform to the behavior of their cannabis’ consuming Friends (22). Based on these studies and our results in the present review, we can conclude that it is important to consider the influences of peers in cannabis prevention efforts.

Although the own popularity was explored by four studies (43, 46–48), results were inconclusive. Those studies, that showed significant positive effects, unfortunately did not control for selection effects (46, 48). The one study that controlled for selection did not find significant effects of popularity (37). Hence, the development of more longitudinal studies controlling for selection effects is needed to study the effects of popularity and possible interactions with popularity on cannabis use.

Regarding the effects of social position on cannabis use, only one study suggested that an adolescent who takes a liaison position in the network could be a potential diffusion agent of behaviors towards cannabis use (43). There is very little evidence to draw any conclusions about the effect of social position on cannabis use. In other research, no association was found between social network position and cannabis use (46). More research is needed to analyze how social positions are related to cannabis use. If different social positions do influence cannabis use, prevention efforts need to consider them as they can possibly interfere in intervention process by empowering (e.g., when multiple non-cannabis users are in a bridge position) or diminishing (e.g., when multiple cannabis users are in a bridge position) intervention effects.

It is also relevant to further explore the changes in peer influence effects over time. One included study showed that age seems to act as a moderator of the peer influence and selection effects on cannabis use (44). For instance, the influence of best friend’s use was greatest at early adolescence, when they are most vulnerable to peer influence. Previous research has shown that at early adolescence, peers become the reference and the main source of support, and adolescents are concerned about the need to feel accepted by those around them (14, 23, 46). This feeling faded when adolescents were older (44, 46, 49).

Our findings showed a large diversity in effects, indicating that more research is needed to clarify peer influence processes on cannabis use. The use of stochastic actor-orientated modelling (SAOM) implies the use of a complete network, as well as leads to a specific and better approach to study peer influence effects controlling for selection effects (47, 50). Data about peer influence on substance use applying SAOM have been published in the last decade, especially for alcohol and tobacco (47, 50). A similar approach is recommended for studying cannabis use.

Some limitations are evident in this review. First, the number of included studies is low, most of them belonged to the Add Health study, and the peer influence are measured differently. Although Add Health is a highly powerful study in terms of social networks, additional research, through other validated surveys, examining social networks in other communities is needed to be able to identify common patterns regarding social networks. Second, the instrument used to assess the methodological quality of this literature does not include questions to assess the control of selection effects, neither the number of adolescents on the network nor its changes over time (i.e., the number of joining and leaving participants in the network). It would be recommended to develop a standard tool to assess the methodological quality of SNA studies.

Despite these limitations, this systematic review supported earlier findings suggesting the role of peer influence on cannabis use among adolescents. An unique point of this review is the specificity and adequacy of the study selection criteria, for instance, including only longitudinal studies and completed social networks.

### 4.1 Implications for future research and practice

Identifying the effect of peer influence on cannabis use is crucial in order to improve the social approach of the traditional socio-cognitive models, as well as prevention and early intervention programs (11–13, 20–23). Once more research would include a broad range of effect in a correct longitudinal design, it could start to translate these peer network influence effects into tools to help intervention programs to become more effective, taking advantage of the composition of the network. Then, it will be of great value to direct prevention efforts towards peer groups that are at risk of cannabis use.

Future research should conduct more longitudinal studies, controlling for selection effects, improving the modelling of the network (i.e., using a complete network), and with more effects of peer influence effects. For instance, studying the role of own popularity and friend popularity, and the influence of the number of alcohol and tobacco friend’s users on cannabis use. It would be recommended that studies control for selection effects. Moreover, as indicated by previous literature (48, 51), it is important to examine more data about the frequency of cannabis use and patterns according to gender and ethnicity, since these could be different in these populations. Additionally, although a growing body of evidence indicates that school environments have a strong influence on adolescent cannabis use (51), future studies could aim to have a broader scope of the network around adolescents, including, for example, out-of-school networks.

It is also important to include direct observations of interactions between targets and peers in order to better understand how peers affect each other and how this influence works overtime. There is already an European intervention trial with social network data for smoking cigarettes (52, 53). Yet, SNA was not yet used within cannabis use prevention, probably due to the lack of adequate SNA studies to support the use of SNA in such programs. Moreover, the European Monitoring Centre for Drugs and Drug Addition has recognized in 2021 the importance of social responses to cannabis-related problems (54). It might be relevant to include the SNA in this type of standards. Finally, further studies may also focus on the impact of the legalization of recreational cannabis use on peer network influence, considering that most of the included articles were conducted before the legalization of marijuana in the USA. The legalization could potentially amplify the positive perception of cannabis use within peer groups.

## 5 Conclusion

To develop effective cannabis prevention programs, it is essential to comprehend the impact of social networks on cannabis use behavior. This systematic review underscores the significance of peer influences on adolescent cannabis use. This review provides a summary of longitudinal findings from scientific evidence regarding peer influence on cannabis use, utilizing the social network approach, and confirming the importance of peer influences on adolescent cannabis use. Different operationalization of the influence of friends’ cannabis use was the most significant peer influence effect on adolescent’s cannabis use. Regrettably, disparities in reporting other peer influence effects hinder optimal comparisons, making it advisable to identify the best methods for measurement in the future (e.g., through consensus meetings or Delphi studies). Therefore, more extensive, and improved research is warranted. Additional studies that control for relevant selection effects are necessary to advance our understanding of these network effects on cannabis use, with an emphasis on expanding the range of network influence effects considered.

## Author contributions

MCTG: Conceptualization, Data curation, Formal analysis, Funding acquisition, Investigation, Methodology, Project administration, Writing – original draft. MABJ: Data curation, Formal analysis, Investigation, Methodology, Supervision, Writing – review & editing. MLS: Conceptualization, Funding acquisition, Resources, Supervision, Writing – review & editing. HV: Conceptualization, Supervision, Writing – review & editing. LM: Conceptualization, Formal analysis, Investigation, Methodology, Supervision, Writing – review & editing.
