# Supplementary material for: The influence of peer’s social networks on adolescent’s cannabis use: a systematic review of longitudinal studies
Source: Front Psychiatry. 2023 Dec 21;14:1306439. doi: 10.3389/fpsyt.2023.1306439 (PMC10768034; doi:10.3389/fpsyt.2023.1306439)
Supplement: Supplementary file 1 [file Table_1.docx]

**Supplementary materials**

Table S1: Definitions of peer influence and peer selection effects on cannabis use.

| **Influence VS selection effect** | **Classification** | **Network’s measures** | **Definitions** |
| --- | --- | --- | --- |
| **Peer network influence effects on cannabis use** | *Endogenous network influence effects* *on cannabis use* | Own popularity^37,40,41^ | Number of received nominations, that is the adolescent is popular themself |
|  |  | The moderating effect of school context (network density, school connectedness, and school level’s alcohol and tobacco use) on the effect of own popularity on cannabis use^40^ |  |
|  |  | Outdegree/out nominations^43,44^ | Number of nominated peers |
|  |  | Neighbourhood density^44^ | Proportion of friendship ties/relationships of an adolescent in relation to the total number of possible ties |
|  |  | Reciprocity^44^ | The tendency to nominate each other as a friend |
|  |  | Network centrality^40^ | The number of ties the respondent has weighted by the number of ties of those to whom he/she sends and receives nominations |
|  |  | Betweenness centrality^44^ | How often an actor is positioned on the shortest path between two other actors |
|  |  | Reach centrality^43,44^ | How close an actor is to all other actors; it can be based on outgoing ties only^37^ and on incoming ties only^38^ |
|  |  | Bonacich power centrality^44^ | Centrality measure that takes into account the centrality of the alters with whom the actor is linked, besides the centrality of ego |
|  |  | Core members^43^ | Adolescent who are multiply connected to the group |
|  |  | Peripheral members^43^ | adolescents who when removing a single friendship tie was sufficient to separate them from the main portion of the group |
|  |  | Liaisons^43^ | Non-group members who had ties to members of two or more groups. Isolates were those participants who had no friendship ties at all or only a single tie to one individual who had no other ties |
|  |  | Other non-members^43^ | Students who were not defined as core, peripheral, isolate, or liaison |
|  |  | Group member^44^ | Those who shared most of their friendship ties with each other and where the removal of one member of the group would not cause the group to be disconnected |
|  |  | Isolate^44^ | Not connected with others within the network |
|  |  | Bridges^44^ | Non-group members with friendship ties to adolescents who are members of different groups, but who were not themselves members of any group |
|  | *Cannabis-related network influence effects on cannabis use* | Friends’ last month cannabis use^37,38,41^ |  |
|  |  | Friends’ lifetime cannabis use^39,42^ |  |
|  |  | Having a best friend cannabis user^44^ |  |
|  |  | Number of neighbourhood cannabis users^44^ |  |
|  |  | Distance to nearest cannabis user considered friends and friend’s friends^44^ |  |
|  |  | Friends’ cannabis use x number of friends^43^ |  |
|  |  | Friends’ cannabis use x reciprocity^41^ |  |
|  |  | Friends’ cannabis use x friend popularity^41^ | *Understanding friend popularity as the popularity of the friends themselves |
|  |  | Friends’ cannabis use x popularity difference^41^ | *Understanding popularity difference as the difference in own popularity and the popularity of the nominated friends |
|  | *Other risk behaviour related network effects on cannabis* | Number of smoking and drinking friends^37^ |  |
|  |  | Peer alcohol and/or tobacco use^40^ |  |
| **Cannabis use related selection effects** | | Ego’s cannabis use^38,39,43,44^ | The effect of an actor’s (ego’s) own cannabis use on selecting other actors |
|  |  | Alter’s cannabis use^38,39,43,44^ | The effect of cannabis use of other Actors (alters) on selection of those alters |
|  |  | Squared alter cannabis use^43^ | The effect of more extreme cannabis use of others on selection of those alters |
|  |  | Same/similar/similarity of cannabis use^37,38,39,41^ | The effect of having similar cannabis use to alters on selection of those alters |

Table S2: A summary overview of peer influence effects.

|  | Wang^37^ | | Schaefer^38^ | De la Haye^39^ | | Vogel40 | Tucker^41^ | | De la Haye^42^ | | | | Osgood^43^ | Ennet^44^ | | |
| --- | --- | --- | --- | --- | --- | --- | --- | --- | --- | --- | --- | --- | --- | --- | --- | --- |
|  | LM CU | | LM CU | LT CU | | LM CU | LM CU | | LM CU | | LT CU | | LM CU | L3M CU | | |
|  | S1 | S2 |  | S1 | S2 |  | S1 | S2 | S1 | S2 | S1 | S2 |  | 11yrs | 13yrs | 15yrs |
| **Endogenous network effects** |  |  |  |  |  |  |  |  |  |  |  |  |  |  |  |  |
| Own popularity | ns | ns |  |  |  | + |  |  |  |  |  |  | ns | + | + | ns |
| Own popularity x density |  |  |  |  |  | ns |  |  |  |  |  |  |  |  |  |  |
| Own popularity x school drug use (alcohol & tobacco) |  |  |  |  |  | ns |  |  |  |  |  |  |  |  |  |  |
| Own popularity x school connect |  |  |  |  |  | - |  |  |  |  |  |  |  |  |  |  |
| Reciprocity |  |  |  |  |  |  |  |  |  |  |  |  |  | ns | ns | ns |
| Neighbourhood density |  |  |  |  |  |  |  |  |  |  |  |  |  | ns | ns | - |
| Outdegree |  |  |  |  |  |  |  |  |  |  |  |  |  | ns | + | + |
| Network centrality |  |  |  |  |  | ns |  |  |  |  |  |  |  |  |  |  |
| Reach centrality |  |  |  |  |  |  |  |  |  |  |  |  | + | + | + | + |
| Betw centrality |  |  |  |  |  |  |  |  |  |  |  |  |  | ns | ns | ns |
| Bonacich power centrality |  |  |  |  |  |  |  |  |  |  |  |  |  | ns | ns | + |
| Bridge group position |  |  |  |  |  |  |  |  |  |  |  |  |  | ns | ns | ns |
| Isolate group position |  |  |  |  |  |  |  |  |  |  |  |  | ns | ns | ns | ns |
| Liaison group position |  |  |  |  |  |  |  |  |  |  |  |  | + |  |  |  |
| Peri member group position |  |  |  |  |  |  |  |  |  |  |  |  | ns |  |  |  |
| Non-member group position |  |  |  |  |  |  |  |  |  |  |  |  | ns |  |  |  |
| **Cannabis related network effects** |  |  |  |  |  |  |  |  |  |  |  |  |  |  |  |  |
| Friend CU | + | + | + |  |  |  | ns | ns | ns | ns | + | ns | + |  |  |  |
| Friend LT CU |  |  |  | + | ns |  |  |  |  |  | ns | ns |  |  |  |  |
| Having a best friend cananbis user |  |  |  |  |  |  |  |  |  |  |  |  |  | + | + | + |
| Number of neighbourhood users |  |  |  |  |  |  |  |  |  |  |  |  |  | + | + | + |
| Distance to nearest cannabis user |  |  |  |  |  |  |  |  |  |  |  |  |  | - | - | - |
| Friend CU x number friends |  |  |  |  |  |  |  |  |  |  |  |  | + |  |  |  |
| Friend CU x reciprocity |  |  |  |  |  |  | + | ns |  |  |  |  |  |  |  |  |
| Friend CU x friend popularity |  |  |  |  |  |  | ns | + |  |  |  |  |  |  |  |  |
| Friend CU x popularity differences |  |  |  |  |  |  | ns | ns |  |  |  |  |  |  |  |  |
| **Other risk behaviour network effects** |  |  |  |  |  |  |  |  |  |  |  |  |  |  |  |  |
| Number of smoking friends | ns | ns |  |  |  |  |  |  |  |  |  |  |  |  |  |  |
| Number of drinking friends | ns | ns |  |  |  |  |  |  |  |  |  |  |  |  |  |  |
| Peer alcohol and/or tobacco use |  |  |  |  |  | + |  |  |  |  |  |  |  |  |  |  |

S=school; LM CU= last month cannabis use; LT CU=lifetime cannabis use; NS=not significant; += positive significant; -= negative significant.
